# Evaluation of Antioxidant and Wound Healing Potentials of *Sphaeranthus amaranthoides* Burm.f.

**DOI:** 10.1155/2013/607109

**Published:** 2013-02-03

**Authors:** R. Geethalakshmi, C. Sakravarthi, T. Kritika, M. Arul Kirubakaran, D. V. L. Sarada

**Affiliations:** Department of Biotechnology, School of Bioengineering, SRM University, Kattankulathur 603203, India

## Abstract

*Background*. *Sphaeranthus amaranthoides* commonly known as sivakaranthai is used in folklore medicine for the treatment of skin diseases. *Methods*. The antioxidant activity of the extract and its fraction was evaluated by using 2, 2-diphenyl-1-picrylhydrazyl (DPPH) free radical scavenging activity, total antioxidant capacity, and total phenolic content. The tested plant extracts showed variable degrees of antioxidant activity. In the present study, methanolic extract of the whole plant of *S. amaranthoides* and a flavonoid fraction obtained from column chromatography were studied for wound healing activity by incorporating the sample in simple ointment base. Wound healing activity was studied in excision wound model in rats, following which, wound contraction, period of epithelization, hydroxyproline content, and collagen levels in the scab were studied. *Results*. Methanolic extract showed the highest antioxidant effect (72.05%) and diethyl ether extract has the least (29.34%) compared to the standard (74.53%). Treatment of wound with ointment containing 5% (w/w) methanolic extract and 5% (w/w) flavonoid fraction exhibited better wound healing activity than positive control (silver sulfadiazine). Finally, histopathology studies conformed wound healing activity in *Sphaeranthus amaranthoides*. The methanolic extract and flavonoid fraction exhibited good wound healing activity probably due to the presence of phenolic and flavonoid constituents. The methanolic extract and flavonoid fraction significantly enhanced the rate of wound contraction and the period of epithelialization comparable to silver sulfadiazine.

## 1. Background

 The genus *Sphaeranthus *in India is represented by three species*, S. indicus, S. africanus *and *S. amaranthoides*. Of the three species, *S. indicus *has been extensively worked [1]. *Sphaeranthus amaranthoides *Burm.f. (Asteraceae) are widely distributed in tropical Asia, Africa, and Australia. *Sphaeranthus amaranthoides *is more effective over *Sphaeranthus indicus. *Ethnomedically, the leaves of *Sphaeranthus amaranthoides *are used by indigenous populations for skin diseases, eczema, acne, dermatitis and wound healing in the form of paste [2]. 

The juice of the plant is styptic and diuretic, and it is said to be useful against liver and gastric disorders. Roots and seeds are used as stomachic and antihelminthic. It has been reported that flowers of this species are highly alterative, depurative, cooling, and tonic. They are also used as blood purifiers in skin diseases [3]. Dried and powdered leaves of *Sphaeranthus amaranthoides *are useful in the treatment of chronic skin diseases, urethral discharges, and jaundice [4]. Extract of *Sphaeranthus amaranthoides *has been reported to exhibit excellent antibacterial activity against Gram-positive as well as Gram-negative bacteria. The phytochemical analysis of the plant showed that it contains steroids, triterpenoids, phenolic compound, flavonoids, tannins, and glycosides. The leaves of *Sphaeranthus amaranthoides* has have been reported for their antioxidant, antimutagenic, antimicrobial, and phytochemical activities [5]. The healing potential of ethanolic extract of aerial parts of *Sphaeranthus amaranthoides *Linn. for treatment of dermal wounds in Wistar rats studied on excision wound models has been reported [6].

The delivery of drug through the skin has long been a promising concept because of the ease of access, large surface area, vast exposure to the circulatory and lymphatic networks, and noninvasive nature of the treatment. Along with other topical dosage forms, herbal drugs are also formulated in the form of ointment. A wound is a break in the normal tissue continuum, resulting in a variety of cellular and molecular sequels. The wound may be created by physical, chemical, thermal, microbial, or immunological, abuse of the tissue [7].

Wound healing is a dynamic process involving biochemical and physiological phenomena that behave in a harmonious way in order to guarantee tissue [8]. The process of wound healing may be hampered by the presence of free radicals, which can damage wound surrounding cells, or by microbial infection [9].

Many herbal plants have a very important role in the process of wound healing. Plants are more potent healers because they promote the repair mechanisms in the natural way. The healing process can be physically monitored by assessing the rate of contraction of the wound [10].

The present study was undertaken to evaluate the antioxidant and wound healing activity of methanolic extract and flavonoid fraction of *Sphaeranthus amaranthoides*. Wound healing activity was evaluated by the observations on wound contraction, wound closure, and collagen formation.

## 2. Materials and Methods

### 2.1. Plant Material

*Sphaeranthus amaranthoides *Burm.f. were collected from Elavanosur Kottai, Villupuram district, Tamil Nadu, India, in December 2010. The plant was taxonomically identified and authenticated by ABS Botanical Conservation, Research and Training Center, Karipatti, Salem (Dt.) (Tamil Nadu), and voucher specimen no. AUT/SRM/06 has been deposited at Plant Tissue Culture Laboratory, SRM University.

### 2.2. Preparation of Plant Extracts

The whole plants of *S. amaranthoides* were washed with running water, air dried, and then chopped into small fragments which are shade dried and reduced to coarse powder with sterile mixer grinder. The powder was passed through a 40-mesh sieve to get the fine powder and stored in an airtight container. The dried powdered plant material (100 g) was successively extracted with 500 mL of diethyl ether (34.6°C), acetone (56°C), chloroform (59.5–61.5°C), methanol (65°C), and water (100°C) for 48 hours in a Soxhlet apparatus. The extracts were then concentrated in rotary vacuum evaporator at reduced pressure below 40°C. The yield of the extract was found to be 3.6, 2.1, 2.4, 15.6, and 5.7%, respectively.

### 2.3. Isolation of Flavonoid Fraction from Methanol Extract

The methanol extract of *Sphaeranthus amaranthoides *(5 gm) was dissolved in minimum quantity of acetone, and it was absorbed over silica gel column (column height: 50 cm, column diameter: 9 cm) of 100–200 mesh. The compounds were eluted with solvents of increasing polarity. Fractions 3–5 (eluted with hexane: ethyl acetate in the ratio of 85 : 15) gave a yellow color liquid with a yield of 750 mg. This fraction gave a positive result for flavonoid. The assay was based on the method of Shinoda [11].

For the animal model studies simple ointments of methanolic extract and flavonoid fraction were formulated in white soft paraffin base at a proportion of 5% (w/w) using a ceramic mortar and pestle.

### 2.4. Animals

Wistar rats weighing 150–250 g were obtained from JIPMER Hospital (Committee for the Purpose of Control and Supervision of Experiments on Animals (CPCSEA) Reg. no. 29/IAEC/10 and no. 30/IAEC/10) Pondicherry registered by Government of India. The animals were housed in a standard individual metal cage and maintained at 22 ± 3°C with an alternating 12 h light-dark cycle. Food and water were provided *ad libitum*.

### 2.5. Pharmacological Evaluation

#### 2.5.1. Antioxidant Activity

*Estimation of Total Phenolic Content (TPC)*. The total phenolics in extracts were determined according to Folin-Ciocalteu procedure of Singleton and Rossi [12]. Four hundred microlitres of the sample (triplicates) were taken in test tubes; 1.0 mL of Folin-Ciocalteu reagent (diluted 10-fold with distilled water) and 0.8 mL of 7.5% sodium carbonate were added, mixed thoroughly, and allowed to stand for 30 min. The absorption was measured at 765 nm against a blank, which contained 400 *µ*L of ethanol. The total phenolic content was expressed as gallic acid equivalents in mg/g of chloroform extract.

#### 2.5.2. DPPH Free Radical Scavenging Activity

The antioxidant activity of the plant extract was estimated using a slight modification of the DPPH radical scavenging protocol given by Chen et al. [13]. For a typical reaction, 2 mL of 100 *µ*M DPPH solution in ethanol was mixed with 2 mL of 100 *µ*g/mL of plant extract. The effective test concentrations of DPPH and the extract were therefore 50 *µ*M and 50 *µ*g/mL, respectively. The reaction mixture was incubated in the dark for 15 min and thereafter the optical density was recorded at 517 nm against the blank. For the control, 2 mL of DPPH solution in ethanol was mixed with 2 mL of ethanol, and the optical density of the solution was recorded after 15 min. The assay was carried out in triplicate. The decrease in optical density of DPPH on the addition of test samples in relation to the control was used to calculate the antioxidant activity, as percentage inhibition (%IP) of DPPH radical:

(1) $$\begin{array}{c} \text{radical}\;\text{scavenging}\;\left(\%\right)\;=\frac{\left({\text{A}}_{\text{control}}-{\text{A}}_{\text{sample}}\right)}{{\text{A}}_{\text{control}}}\times 100. \end{array}$$

#### 2.5.3. Wound Healing Activity

*Excision Wound Model*. For excision wound study, male Wistar rats (150–250 g) were selected and divided into four groups of six animals each. Rats were anaesthetized with anesthetic ether and depilated at the predetermined site before wounding. An excision wound was inflicted by cutting away approximately 500 mm^2^ full thickness of the predetermined area on the anterior-dorsal side of each rat [14].

Animals were treated by external application of different formulations at a final concentration of 10 mg/Kg body weight. Group I animals were treated with methanolic extract paste (5% w/w), Group II animals were treated with flavonoid fraction paste,  Group III (control group) animals received base ointment alone, and Group IV animals (positive control group) received silver sulfadiazine. Test formulations were applied to respective groups twice a day for 10 days starting from the day of wounding.

Wound healing property was evaluated by wound contraction percentage and closure time. The wound area was measured every third day by placing a transparent paper over the wound and tracing it out; area of this impression was calculated using the graph sheet [15], and wound contraction was expressed as percentage of contraction. Wound closure time was recorded when total wound healed. Further hydroxyproline and collagen levels were estimated at the beginning and the end of the experiment.

#### 2.5.4. Hydroxyproline Estimation

On day 1 and day 10, a piece of skin from the wound area was collected and analyzed for hydroxyproline content, which is a basic constituent of collagen. The tissue sample is dried in a hot air oven at 60°C for 12 h and weighed to determine the dry granulation tissue. Dried granulation tissues are added with 6 N hydrochloric acid (1.0 mL for 100 mg of sample) and then kept at 110°C for 24 h in sealed tubes. 1.0 mL of acid hydrolysate sample is mixed with 1 mL each of 0.01 M copper sulfate solution, 2.5 N sodium hydroxide, and 6% hydrogen peroxide. The solutions are mixed and incubated at 80°C for 5 minutes. The tubes are chilled in an ice water bath, and 4 mL of 3 N sulfuric acid were added with agitation followed by addition of 2 mL of p-dimethylaminobenzaldehyde (5%) solution and mixed thoroughly. The tubes were placed in a water bath at 70°C for 15 minutes and then chilled in an ice water bath. The absorbance was measured at 540 nm [16].

#### 2.5.5. Collagen Estimation

On day 1 and day 10, a piece of skin from the wound area was collected and analyzed for collagen content. 5 mL of TES buffer and 0.1 mL of acid hydrolysate sample was added in the test tube, mix well and incubated at 37°C. After 5 hours, the contents were filtered through a syringe filter (0.8 *µ*M) into clean containers and allowed to stand. For color development 0.2 mL of test filtrate and 2 mL of ninhydrin color Reagent (NCR) were added, mixed well, and placed in a boiling water bath for 30 minutes. After cooling to room temperature 10 mL of 1-propanol was added to each container, mixed well, and the absorbance was read at 570 nm [17, 18].

#### 2.5.6. Histopathological Studies

Skin specimens from rats of 4 groups were collected in 10% buffered formalin, and after the usual processing, 5 *µ*m thick sections were cut and stained with hematoxylin and eosin [19]. Sections were qualitatively assessed under the light microscope and graded with respect to fibroblast proliferation, collagen formation, epithelisation, and keratinisation.

#### 2.5.7. Statistical Analysis

The results were expressed as mean ± SD. Data were analyzed by one-way analysis of variance (ANOVA) followed by the Tukey's test for multiple comparisons.

## 3. Results

### 3.1. Antioxidant Activity

#### 3.1.1. Total Phenolic Content

The total phenolic content expressed as GAE (gallic acid equivalents) was recorded to be high in the methanolic extract (2.15 ± 0.26 mg/g dry weight) among the four different extracts studied while the lowest values were recorded in diethyl ether extract (0.63 ± 0.25 mg/g dry weight).

### 3.2. Antioxidant Activity (DPPH Free Radical Scavenging)

Percentage of inhibition calculated by the comparison of control and test samples for the determination of antioxidant activity revealed the highest value for methanol extract (72.05 ± 0.12 mg/mL) which was also comparable to butylated hydroxyl anisole (74.53 ± 0.06 mg/mL) followed by chloroform and acetone extracts (52.02 ± 0.09, 56.65 ± 0.9 mg/mL) (values are mean ± SD of triplicates).

### 3.3. Wound Healing Activity

#### 3.3.1. Excision Model

**Figure 1:**
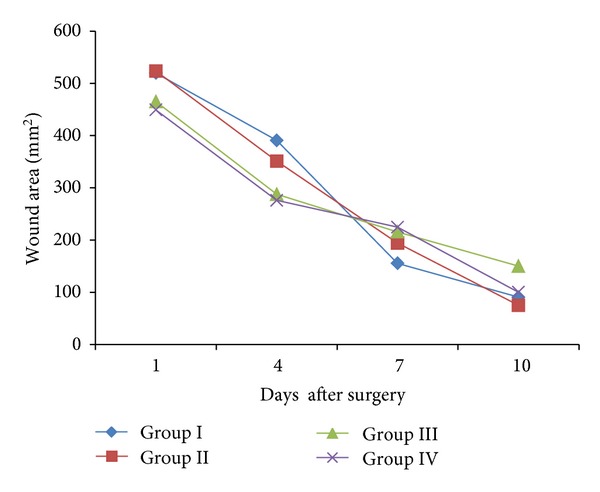
Comparative mean wound area of different experimental animal groups recorded on different days after surgery (values are mean ± SD of six values).

The area of wound measured on the days 1, 4, 7, and 10 after surgery in all groups revealed the following interesting results. A very rapid closure of the wound in both extract-and flavonoid fraction-treated groups observed between 7 and 10 days after surgery. After day 7 of surgery, wound closure was rapid, and total closure of the wound was observed by the 10th day. These results were comparable to the group treated with silver sulfadiazine. The control group, treated with simple ointment, showed very little contraction compared with methanol extract and flavonoid fraction (Figure 1).

#### 3.3.2. Hydroxyproline Estimation

**Figure 2:**
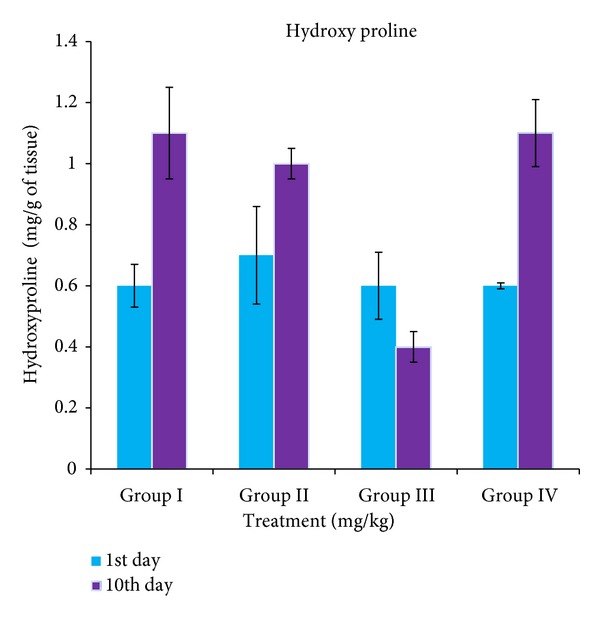
Variations in hydroxyproline concentrations in different experimental animal groups.

Hydroxyproline levels were found to be increased significantly in the groups treated with extract, flavonoid fraction, and standard sulfadiazine (1.1 ± 0.15, 1.0 ± 0.05, and 1.1 ± 0.11 mg/g of tissue, resp.) than the control group (0.4 ± 0.05 mg/g of tissue) which implies more collagen deposition in treated groups than the control group (Figure 2). Significant differences were observed in hydroxyproline concentrations between groups 2 and 3 when compared with control group 4 with *P < 0.01.

#### 3.3.3. Collagen Estimation

In our present study, collagen level of wounded region was found to be increased in the groups treated with flavonoid fraction, extract, and standard sulfadiazine (0.96 ± 0.02, 0.61 ± 0.02, and 0.82 ± 0.05 mg/g of tissue, resp.) against negative control (0.33 ± 0.04 mg/g of tissue) which implies more collagen deposition in treated groups than the control group (Table 1). Significant differences were observed in collagen concentrations between groups 2 and 3 when compared to control group 4 with *P < 0.01.

### 3.4. Histopathological Observations

**Figure 3:**
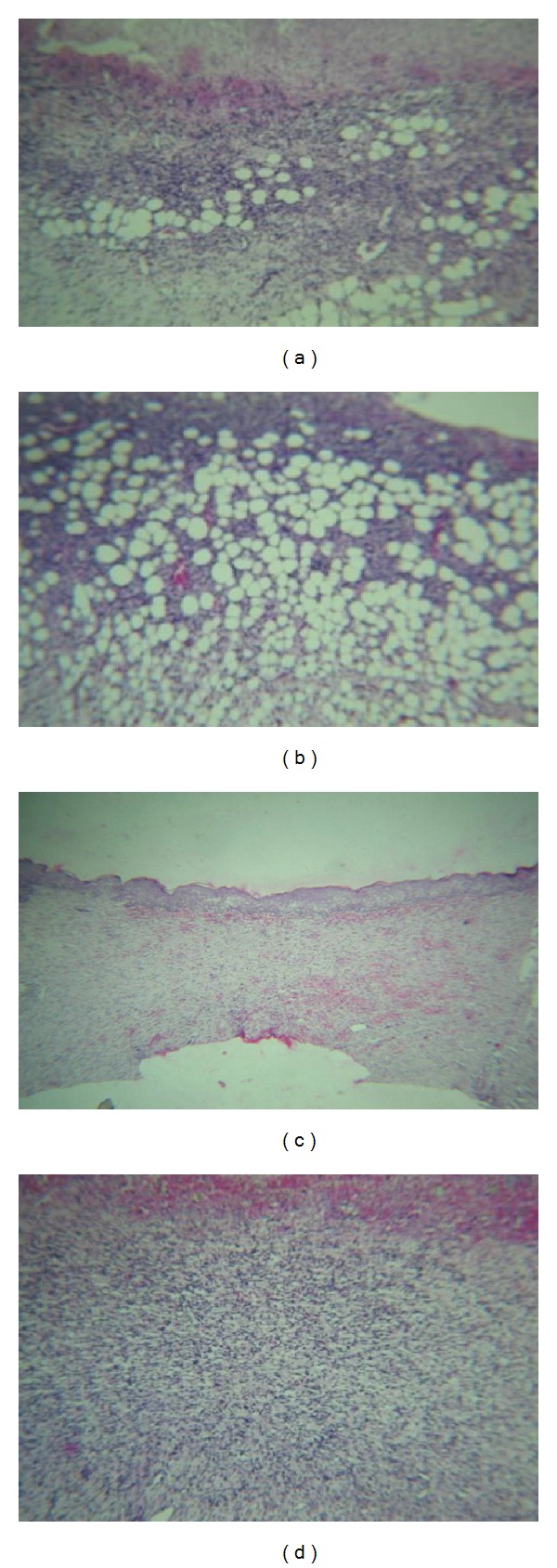
Hematoxylin and eosin (HE) stained skin sections ((a)–(d)) of tissues from the healed area of the wound of animals from different groups treated with extract and fractions from *S. amaranthoides* at 10x magnification. (a) Ulcer in the skin section of rat treated with methanol extract of *S. amaranthoides* showing moderate amount of grade II granulation tissue beneath the blood clot and fibrin clot. (b) Minimal amount of vascular granulation tissue of grade I in the skin section treated with flavonoid fraction of *S. amaranthoides.* (c) Complete reepithelisation with no ulceration in vehicle control group. (d) Vascular granulation tissue of grade II with proliferating fibroblasts in the rat skin section treated with standard silver sulfadiazine.

Treatment of rat wounds with methanol extract and flavonoid fraction ointments has led to enhanced fibroblast proliferation, epithelisation as compared to vehicle-treated group or control group. Photographs of skin sections representing these features are presented in Figure 3.

## 4. Discussion

The medicinal properties of plants have been the centre of attraction for researchers in recent scientific developments throughout the world, due to their potent antioxidant properties and economic viability [20].

DPPH assay is a convenient, accurate, and easy method, and therefore, it is widely used to measure the antioxidant activity of plant extracts. Under the assay conditions employed here, the methanolic extract showed higher antioxidant activity while comparing to other extracts. Positive correlation has been demonstrated between antioxidant activity and phenolic content of plant extracts [21].

Wound healing is a very complex, multifactor sequence of events involving several cellular and biochemical processes. The aims in these processes are to regenerate and reconstruct the disrupted anatomical continuity and functional status of the skin [22]. Healing process, a natural body reaction to injury, initiates immediately after wounding and occurs in four stages. The first phase is coagulation, which controls excessive blood loss from the damaged vessels. The next stage of the healing process is inflammation and debridement of the wound followed by reepithelialization, which includes proliferation, migration, and differentiation of squamous epithelial cells of the epidermis. In the final stage of the healing process collagen deposition and remodeling occurs within the dermis [23].

Wound contraction is the process of mobilizing healthy skin surrounding the wound to cover the denude area. This centripetal movement of wound margin is believed to be due to the activity of myofibroblast. In recent years, oxidative stress has been implicated in a variety of degenerative processes and diseases; these include acute and chronic inflammatory conditions such as wound [24].

Reactive oxygen species are involved in a number of degenerative diseases such as atherosclerosis, cancer, cirrhosis, and diabetes including wound healing [25]. Antioxidants enhance the healing of infected and noninfected wounds by reducing the damage caused by oxygen radicals [26]. Plant-derived antioxidants such as tannins, lignans, stilbenes, coumarins, quinones, xanthones, phenolic acids, flavones, flavonols, catechins, anthocyanins, and proanthocyanins could delay or prevent the onset of degenerative diseases because of their redox properties, which allow them to act as hydrogen donors, reducing agents, hydroxyl radicals (OH), or superoxide radical (O_2_) scavengers. In the present study antioxidant activity levels were found to be relatively high in the methanolic extract and flavonoid fraction; further the process of wound healing was also enhanced in the animals treated with the methanolic extract and flavonoid fraction indicating a clear role of these constituents in the process of wound healing.

The inflammation stage begins immediately after injury, first with vasoconstriction that favors homeostasis and releases inflammation mediators. The proliferative phase is characterized by granulation tissue proliferation formed mainly by fibroblast and the angiogenesis process. The remodeling stage is characterized by reformulations and improvements in the components of the collagen fiber that increases the tensile strength [27]. Wound healing is a process by which a damaged tissue is restored as closely as possible to its normal state, and wound contraction is the process of shrinkage of area of the wound. It depends upon the reparative abilities of the tissue, type and extent of the damage, and general state of the health of the tissue. The granulation tissue of the wound is primarily composed of fibroblast, collagen, edema, and small new blood vessel [28].

The course of searching an ethnopharmacologically active plant extract down to a single active principal may result in a defeat of biological activity for a number of reasons; for instance, a special compound might be unstable during extraction, fractionation, or in the purified form or the fundamental basis for ethnopharmacology does not always exist in a single compound but rather is a result of the interaction of more than one active compound found in the extract [29].

Ulcer in the skin section of the rat treated with methanol extract of *S. amaranthoides* showed moderate amount of grade II granulation tissue beneath the blood clot and fibrin clot. Minimal amount of vascular granulation tissue of grade I showed in the skin section treated with flavonoid fraction. The undifferentiated mesenchymal cells of the wound margin modulate themselves into fibroblast, which start migrating into the wound gap along with the fibrin strands. The collagen is the major component of extracellular tissue, which gives support and strength, and is composed of hydroxyproline amino acid [30]. Collagen level was found to be increased in flavonoid fraction as 0.96  ±  0.02 mg/g of tissue, and hydroxyproline concentration was also gradually increased as 1.1 ± 0.65 mg/g of tissue.

The results in this study are in support that the wound healing and repair is accelerated by applying extracts and flavonoid fraction of *Sphaeranthus amaranthoides* which is highlighted by the full thickness coverage of the wound area by an organized epidermis in the presence of mature scar tissue in the dermis. This ability was especially obvious when the data were compared with the other plants. A cream containing ethanolic extract of aerial parts of *Sphaeranthus indicus*, Linn. (Asteraceae) was evaluated for wound healing activity in guinea pigs. The cream was applied in vivo on the paravertebral area of six excised wounded models once a day for 15 days. The cream significantly enhanced the rate of wound contraction and the period of epithelialization comparable to neomycin [31].

The enhanced capacity of wound healing with the *Sphaeranthus amaranthoides *could be explained on the basis of the anti-inflammatory effects as documented in the literature.

In the wound healing studies, the wound closure time and wound contraction were taken as parameters. In both methanolic extract and flavonoid fraction wound closure time was 10 days. As shown in Figure 1, around 90% of wound healing was recorded in both the treated groups, but only 65% was recorded in control group on the 10th day after surgery. Collagen formation and concentration both will affect the breaking strength of the skin. Results obtained in this study confirm the wound healing activity of *Sphaeranthus amaranthoides*.

## 5. Conclusion

In conclusion, while plant-based traditional medicine has been used throughout generations, the efficacy of such treatments requires experimental backup and scientific verification. In this study, *Sphaeranthus amaranthoides* was selected based on ethnopharmacological information, provided by the local communities.* Sphaeranthus amaranthoides *has a positive influence on the collagen content and stability in a wound and therefore a beneficial role in wound healing; further the synergistic effect of antioxidant activity accelerated the wound healing process.

## Figures and Tables

**Table 1:** Collagen level on the 1st and the 10th days in different experimental animal groups.

Groups	Treatment	Collagen (mg of leucine/g of tissue)	
		1st day	10th day
1	Methanol extract	0.21 ± 0.04	0.61 ± 0.02*
2	Flavonoid fraction	0.22 ± 0.03	0.96 ± 0.02*
3	Ointment control	0.22 ± 0.02	0.33 ± 0.04
4	Silver sulfadiazine	0.25 ± 0.03	0.82 ± 0.05*

Values are mean ± SD, significant when compared to control.

*P < 0.01 in all values.
